# Ultradian oscillations and pulses: coordinating cellular responses and cell fate decisions

**DOI:** 10.1242/dev.104497

**Published:** 2014-10

**Authors:** Akihiro Isomura, Ryoichiro Kageyama

**Affiliations:** 1 Institute for Virus Research, Kyoto University, Shogoin-Kawahara, Sakyo-ku, Kyoto 606-8507, Japan; 2 Japan Science and Technology Agency, Core Research for Evolutional Science and Technology (CREST), 4-1-8 Honcho, Kawaguchi, Saitama 332-0012, Japan; 3 World Premier International Research Initiative–Institute for Integrated Cell-Material Sciences (WPI-iCeMS), Kyoto University, Kyoto 606-8501, Japan

**Keywords:** Negative feedback, Optogenetics, Ultradian oscillator, Systems biology

## Abstract

Biological clocks play key roles in organismal development, homeostasis and function. In recent years, much work has focused on circadian clocks, but emerging studies have highlighted the existence of ultradian oscillators – those with a much shorter periodicity than 24 h. Accumulating evidence, together with recently developed optogenetic approaches, suggests that such ultradian oscillators play important roles during cell fate decisions, and analyzing the functional links between ultradian oscillation and cell fate determination will contribute to a deeper understanding of the design principle of developing embryos. In this Review, we discuss the mechanisms of ultradian oscillatory dynamics and introduce examples of ultradian oscillators in various biological contexts. We also discuss how optogenetic technology has been used to elucidate the biological significance of ultradian oscillations.

## Introduction

The temporal coordination of gene function is essential for the precise control of cellular activity and function in living organs. Cell cycle and circadian oscillators are well-conserved molecular machineries that are found in many species. Such biological clocks generate troughs and ridges in temporal patterns of biochemical activities, and they maintain a periodicity of about 10 to 24 h, thus confirming adaptation to daily light and growth signals. However, recent progress in molecular and cellular biology has revealed yet another class of oscillators, called ultradian oscillators, which tick with a much shorter periodicity than 24 h, typically ranging from 2 to 4 h in mammalian cells. Although they relate to diverse biological phenomena, such as developmental processes, cell proliferation, DNA damage responses and immune responses, the precise roles and importance of these ultradian oscillations or pulses are still controversial.

In this Review, we provide an overview of the molecular and theoretical basis of cellular oscillators. We also present examples of ultradian oscillators that are found in various biological contexts in mammals. We then discuss how the artificial control of temporal cellular activities has been applied to elucidate the functional outcomes of ultradian oscillations. In particular, recent progress using optogenetic technology is summarized, highlighting how this approach can be used to reveal the biological significance of ultradian oscillations in controlling cellular responses and determining cell fates.

## The basis of cellular oscillators: theory and molecular architectures

Gene regulatory networks play a central role in developmental processes and lead to the dynamic spatio-temporal pattern of gene expression. Gene expression levels are regulated mainly by transcription factors, but the abundance and activities of these transcription factors are in turn regulated by other types of proteins, such as kinases and phosphatases. The expression levels of these regulatory proteins are also under the control of gene regulatory networks, meaning that multiple genes functionally interact with each other. The orchestration of various kinds of network dynamics in individual cells thus results in time-dependent morphological changes in developing embryos and gives rise to a rich variety of structures of living organs. Hence, understanding the regulatory mechanisms of gene expression dynamics is a crucial step to elucidate the design principle of developmental tissue formation.

**Fig. 1.**
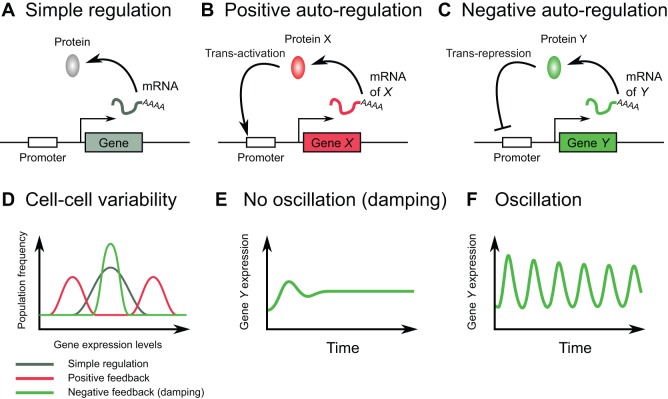
**Gene regulatory network motifs and gene expression dynamics.** (A) Simple regulation. In this case, a gene is transcribed and the resultant mRNA is translated into protein, which eventually turns over. (B) In the case of positive auto-regulation, the gene product activates its own expression. (C) In negative auto-regulation, the gene product represses its own production. (D) Schematic illustration of cell-cell variability in gene expression levels in the case of simple, positive auto- and negative auto-regulation. A positive-feedback loop can generate two states, whereas a negative-feedback loop without time delay can decrease the cell-cell variability compared with that generated by a simple regulatory circuit (Alon, 2007). (E,F) Temporal patterns of negative-feedback circuits with different parameters. For example, a short delay in a negative-feedback loop leads to dampened oscillations (E), but if the appropriate parameters are satisfied, oscillation is maintained (F).

Gene regulatory networks typically involve feedback, feed-forward loops and auto-regulatory circuits, which are called network motifs (Fig. 1A-C). Some motifs often appear in networks of various species and generate gene expression patterns in a spatio-temporal manner (Alon, 2007). For instance, positive-feedback loops are known to constitute a toggle-switch of gene expression control, which works as a switch for cell fate determination (Tyson et al., 2003; Eldar and Elowitz, 2010). The simplest positive-feedback loop is positive auto-regulation (Fig. 1B), whereby a transcriptional activator (here, X) initiates its own expression; an example of such a transcriptional activator is the myogenic determination factor MyoD1 (Thayer et al., 1989). This molecular machinery is useful to generate sub-populations of cells, e.g. X-high and X-low, that exhibit distinct expression levels and increased cell-cell variability (Fig. 1B,D). Furthermore, if X is a master gene of cell fate determination, the X-high and X-low sub-populations might correspond to differentiated and undifferentiated cells, respectively.

A negative-feedback loop is another typical example of a network motif that also appears in a number of biological contexts. Negative autoregulation (Fig. 1C) is one of the simplest cases of a feedback loop, whereby a transcriptional repressor (here, Y) binds to its own promoter region and inhibits its own gene expression. One characteristic of negative autoregulation without time delay is the short time required to reach steady-state (saturated) levels of gene expression in response to external stimuli (Fig. 1C-E) when compared with simple regulation and positive autoregulation (Fig. 1A,B,D), indicating an advantage of negative autoregulation for environmental adaptation. However, in some conditions, the expression levels of negative autoregulators are not sustained but instead fluctuate in an oscillatory manner (Fig. 1F), which is a cyclic sequence of the following events: (1) synthesis of the gene product Y; (2) repression of new synthesis of Y by accumulating Y; (3) degradation of Y; and (4) re-synthesis of Y, thereby starting the next cycle of the oscillation. Such oscillatory expression driven by negative autoregulation is likely to be one of the important modes of gene regulation and can be found in a number of biological events. However, although negative feedback is an essential mechanism for oscillations, it is insufficient for their emergence (Tyson et al., 2003; Alon, 2007; Novak and Tyson, 2008). There are additional required conditions, which are related to the kinetic parameters of molecular turnover and the network structures of feedback loops (Lewis, 2003; Monk, 2003; Novak and Tyson, 2008). For example, it has been suggested that negative autoregulation with appropriate delays is required for oscillatory expression (Jensen et al., 2003; Lewis, 2003; Monk, 2003). There are a number of mechanisms that can generate such delays in negative-feedback loops, such as increasing the non-linearity of chemical reactions or increasing the number of cascades in the loops (Novak and Tyson, 2008). One striking proof of the latter case has been exemplified by the generation of synthetic oscillators using three repressor proteins (Elowitz and Leibler, 2000). In a simple negative autoregulation system, depending on the intensity of stimulus, the amplitude of oscillatory expression changes, but its frequency does not.

Other examples of oscillation are based on the combination of a positive-feedback loop with a negative-feedback loop, which also contributes to the generation of time delay (Tyson et al., 2003; Novak and Tyson, 2008; Stricker et al., 2008; Tigges et al., 2009). When a positive-feedback loop is followed by slow negative feedback, the network forms an excitable system with a delayed refractory period, similar to the electro-physiological activity observed in nerve cells, which generates transient pulsatile dynamics upon external stimulation. In this case, a continuous stimulus gives rise to repeating pulses with fixed amplitudes, but the frequency of pulses changes dependent on the noise intensity. In general, it is difficult to define a clear border between such pulsatile dynamics and negative autoregulation-driven oscillation, especially in experimental measurements. Because of this difficulty, we do not differentiate between oscillation and pulsatile dynamics in this Review and, for the sake of simplicity, we refer to ‘oscillations’ and ‘pulses’ in the same context.

## The segmentation clock: a case study for understanding ultradian oscillators

The segmentation clock, which regulates the periodicity of somite formation, involves a number of molecular oscillators and hence has provided many insights into the roles and regulation of ultradian oscillators (Dequeant and Pourquie, 2008; Oates et al., 2012; Kageyama et al., 2012). Hes7, for example, which is expressed in an oscillatory manner in the presomitic mesoderm (PSM, the tissue that gives rise to the somites), is a basic helix-loop-helix (bHLH) transcriptional repressor that suppresses its own transcription by interacting with its promoter. Hes7 is known to act as one of the core molecular clock factors during somite formation in developing mouse embryos, generating 2 h-period oscillations with fast (i.e. a half-life of about 20 min) mRNA and protein turnover rates (Bessho et al., 2001, 2003). Such kinetic parameters of molecular turnover are crucial for ultradian oscillations. To address the importance of this turnover, Hirata et al*.* analyzed a mutant form of Hes7 that exhibits a longer half-life (about 30 min) than the wild-type protein (about 20 min) but displays normal repressor activity (Hirata et al., 2004). They generated knock-in mice that express the mutant Hes7 and found that somite formation was severely disrupted after a few normal cycles of segmentation. As the Hes7 negative-feedback circuit consists of a small number of components, the oscillatory dynamics can be reproduced by a simple mathematical model (Jensen et al., 2003; Lewis, 2003; Monk, 2003; Novak and Tyson, 2008). Such mathematical simulations with altered parameters for protein half-life could reproduce the effect of Hes7 protein stabilization, supporting the importance of a rate-constant for protein turnover in the segmentation clock (Hirata et al., 2004).

The half-life of mRNA also plays an essential role in the maintenance of oscillation. Hes1, which also oscillates during somitogenesis, is another bHLH repressor protein that represses its own expression. Due to this negative feedback, Hes1 expression oscillates with a 2- to 3-h periodicity, and these oscillations are observed in a variety of other cell types, including fibroblasts, neural progenitors and embryonic stem (ES) cells (Hirata et al., 2002; Masamizu et al., 2006; Kageyama et al., 2007; Shimojo et al., 2008; Kobayashi et al., 2009; Imayoshi et al., 2013). In many cell types, the half-life of *Hes1* mRNA is about 20 min, but in mouse ES cells it is about 40 min (Kobayashi et al., 2009). Interestingly, in mouse ES cells, the period of *Hes1* oscillation is also longer (about 4 h), highlighting the importance of mRNA turnover for tuning the oscillation period. Moreover, the stabilization of *Hes1* mRNA half-life by knockdown of micro-RNA 9 (miR-9), which is complementary to the 3′-UTR sequence of *Hes1* mRNA, disrupted oscillations in neural stem and progenitor cells (Tan et al., 2012; Bonev et al., 2012). These results suggest a functional role for mRNA stability in the regulation of oscillatory dynamics.

Another key factor that can influence oscillations is a delay in the time required to complete the negative-feedback loop. The negative autoregulation of *Hes7* involves several processes, including transcription of the exon and intron sequences, maturation of the RNA by splicing of intronic sequences, export of mRNA from the nucleus to the cytosol, translation of the protein, protein binding and, finally, the repression of transcription. If these sequential processes are finished too quickly, giving rise to a short delay period, the system can reach a steady state. To understand the significance of a delay in the negative-feedback loop, Takashima et al*.* examined whether the intronic delay, which is the time necessary to transcribe and splice out intron sequences to generate mRNAs, is essential for the stable oscillations of Hes7 in the PSM (Takashima et al., 2011). They generated mutant mice lacking the intron sequences of *Hes7* gene alleles, and found that the oscillatory expression of Hes7 is abolished in these mice, resulting in severe fusion of somites. This experimental result was recapitulated by a mathematical model based on the delayed negative-feedback loop (Lewis, 2003; Monk, 2003). Further investigation of the mathematical model with parameter tuning predicted that moderate shortening of the intronic delay results in accelerated (i.e. a shorter period of) but dampened oscillation. Harima et al. further examined this prediction by generating transgenic mice harboring various combinations of intronic sequences of *Hes7* (Harima et al., 2013). Mutant mice that retained only the third intron within the *Hes7* gene showed an accelerated tempo of the segmentation clock in the anterior region and an increased number of cervical vertebrae (nine cervical vertebrate compared with seven in the wild type) but fusion of the posterior somites. It is worth noting that these introns are present not only in the mouse *Hes7* gene but also in the zebrafish and chick homologs (Hoyle and Ish-Horowicz, 2013), indicating that the intronic delay is a basic and conserved mechanism that stabilizes the segmentation clock in vertebrate embryos. Moreover, intronic delay appears in other biological contexts, such as in the TNF-induced inflammation process, in which the expression of various genes occurs at different timings due to different speeds of the splicing events (Hao and Baltimore, 2013). It has also been reported that intronic delay can contribute to the generation of synthetic genetic oscillations (Swinburne et al., 2008). Thus, there might be more situations in which intronic delays play important roles.

In summary, these examples of oscillations during the segmentation clock demonstrate how oscillatory gene expression can be generated and how it can be modified by various parameters.

## Other examples of ultradian oscillators: oscillatory and sustained dynamics lead to different outcomes

The segmentation clock has provided various insights into molecular oscillators but it is becoming evident that such oscillators also exist in other contexts. The recent development of live cell-imaging techniques, such as those using fluorescent probes and bioluminescence reporters, has enabled the analysis of various types of dynamic biochemical activities and has revealed ultradian oscillations at single-cell levels (Levine et al., 2013; Purvis and Lahav, 2013). These studies suggest an increasing number of such examples in mammalian cells: pulsatile activities of phosphorylated extracellular signal-regulated kinase (ERK) in cell proliferation (Sasagawa et al., 2005; Shankaran et al., 2009; Albeck et al., 2013; Aoki et al., 2013), oscillatory dynamics of the tumor suppressor protein p53 upon DNA damage (Lahav et al., 2004; Geva-Zatorsky et al., 2006; Loewer et al., 2010; Batchelor et al., 2011; Purvis et al., 2012), oscillations in NF-κB expression during immune responses (Hoffmann et al., 2002; Nelson et al., 2004; Ashall et al., 2009; Tay et al., 2010) and oscillations of the Notch effector protein Hes1 during cell differentiation (Shimojo et al., 2008; Kobayashi et al., 2009; Imayoshi et al., 2013). Below, we discuss each of these examples in turn, highlighting the possible biological relevance of these molecular oscillators.

### Hes1 and neural differentiation

**Fig. 2.**
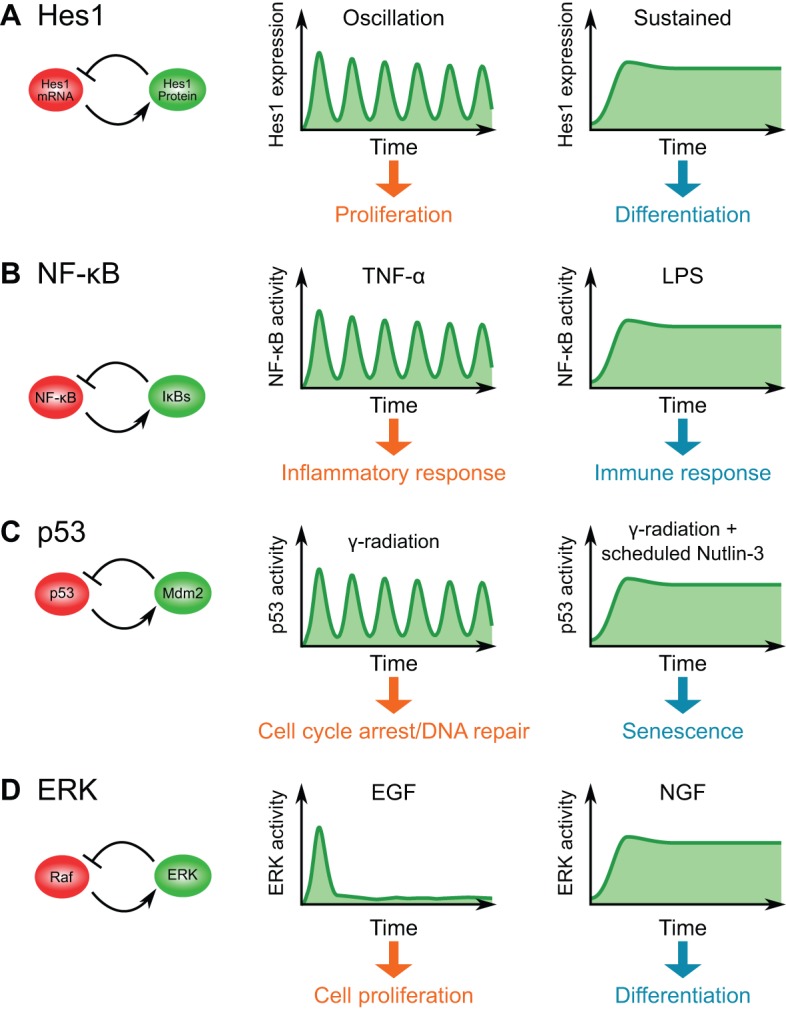
**Ultradian oscillators in various biological contexts.** (A) Hes1 oscillation. (B) NF-κB oscillation. (C) p53 oscillation. (D) Extracellular signal-regulated kinase (ERK) pulse generation. For each case, the negative-feedback motif (left) and the relationship between the expression dynamics and the biological outcomes (right) are shown. Note that the negative feedback shown in the case of ERK/Raf is hypothetical.

In addition to its role during somitogenesis, Hes1 regulates the proliferation and differentiation of stem/progenitor cells in various developmental contexts. For example, in proliferating neural progenitor cells, which are multipotent and have the potential to generate neurons, astrocytes and oligodendrocytes, Hes1 expression oscillates (Fig. 2A) (Shimojo et al., 2008). However, Hes1 expression becomes sustained when neural progenitor cells differentiate into astrocytes (Fig. 2A) (Imayoshi et al., 2013). Thus, the dynamics of Hes1 expression are different in different contexts.

Similarly, the expression dynamics of the proneural factor Ascl1 are different between proliferating and differentiating cells. Hes1 represses Ascl1 expression, and Hes1 oscillations thereby drive Ascl1 oscillations in neural progenitors (Imayoshi et al., 2013). In differentiating neurons, however, Hes1 expression disappears, leading to sustained expression of Ascl1, which induces neuronal differentiation. This temporal switching from oscillatory to sustained patterns of Ascl1 upon exposure to external differentiation cues has been successfully visualized at single-cell level by bioluminescence imaging of luciferase reporters, thus raising the possibility that the dynamic expression of a single transcription factor, Ascl1, generates two distinct states, proliferation or differentiation (Imayoshi et al., 2013).

### NF-κB oscillations and the inflammatory response

Ultradian oscillations have also been observed in the NF-κB signaling pathway, which regulates the response to pathogens and stress (Oeckinghaus et al., 2011). Members of the NF-κB family of transcription factors usually form dimers such as the RelA/p65 complex. These NF-κB complexes associate with IκB inhibitory proteins and are localized in the cytoplasm in the absence of external stimulation because IκBs mask the NF-κB nuclear localization signals. However, upon stimulation by factors such as tumor necrosis factor α (TNFα), the IκB kinase (IKK) is activated and initiates proteasomal degradation of IκBs, thereby allowing the NF-κB complexes to enter the nucleus, bind to DNA and activate the expression of target genes, including their own inhibitor, IκBα. The newly synthesized IκBα binds to NF-κB and leads to re-inhibition of NF-κB by triggering its export to cytoplasm, thus forming a negative-feedback loop (Hoffmann et al., 2002; Nelson et al., 2004; Kearns et al., 2006; Ashall et al., 2009; Tay et al., 2010). Due to the rapid degradation of IκBs and the time delay between their subsequent transcription and translation, ultradian oscillations in NF-κB signaling emerge (Fig. 2B). These oscillations can be visualized at single-cell level by means of fluorescent fusion reporters, such as RelA-DsRed fusions (Nelson et al., 2004; Tay et al., 2010). The expression of RelA-DsRed proteins at nearly physiological amounts mimics the nuclear-cytoplasmic (N-C) shuttling of endogenous RelA proteins and enables the level of NF-κB signaling to be quantified based on the N-C ratio of red fluorescence.

What is the functional role of NF-κB oscillation? In attempt to answer this question, Ashall et al. applied TNFα to populations of RelA-DsRed-expressing cells for various periods (60, 100 or 200 min) or in a continuous fashion (Ashall et al., 2009). They found that N-C translocations of RelA-DsRed were synchronous at a single-cell level when stimulated by 200 min interval pulses, but asynchronous under other conditions (with 60 or 100 min periodicity, or continuously), indicating that the oscillation period controls the type of cellular response. Western blot analysis and quantitative polymerase chain reaction (qPCR) analysis further revealed that different schedules of TNFα exposure activate different sets of genes. Early response genes are rapidly activated by both transient stimulation and continuous stimuli, whereas gene products of late response genes gradually accumulate only when stimuli are continuously delivered (Ashall et al., 2009; Tay et al., 2010). One key parameter for determining early and late response genes is the kinetics of the gene products, such as mRNA half-life and intronic delay (Hao and Baltimore, 2013). On the other hand, lipopolysaccharide (LPS) induced slow activation of NF-κB signaling, leading to a different gene expression program (Fig. 2B) (Werner et al., 2005). Together, these data suggest that the dynamics of NF-κB oscillation determines which genes are activated, leading to different cellular responses.

### Oscillatory p53: determining cell cycle arrest, senescence and apoptosis

The tumor suppressor protein p53 is also known to operate as an ultradian oscillator (Lev Bar-Or et al., 2000; Lahav et al., 2004; Geva-Zatorsky et al., 2006; Loewer et al., 2010; Batchelor et al., 2011; Purvis et al., 2012). Upon γ-irradiation, double-strand DNA breaks are induced in cells, and this activates the kinases ataxia telengiectesia mutated (Atm) and checkpoint kinase 2 (Chk2), which in turn phosphorylate p53. Phosphorylated p53 is stabilized and accumulates in the nucleus, where it induces the transcription of a number of target genes, including its own positive and negative regulators. One major inhibitor of p53 is an E3 ubiquitin ligase, mouse double minute 2 (Mdm2), which facilitates the proteasomal degradation of p53 proteins and contributes to a negative-feedback loop. As Mdm2 is also degraded rapidly (with a half-life of 30 min), oscillatory patterns of p53 activity appear with a period of about 4 h (Fig. 2C) (Lahav et al., 2004; Geva-Zatorsky et al., 2006). In unstimulated cells, temporal patterns of p53 activity are observed as transient pulses and in a random fashion, indicating an excitable mechanism as a pulse generator (Loewer et al., 2010).

When sustained induction of p53 activity was artificially stimulated by a temporally scheduled dose of the small molecule Nutlin-3, which inhibits Mdm2, cells chose the fate of senescence rather than apoptosis (Fig. 2C), suggesting that p53 oscillation functions as a cell fate determinant (Purvis et al., 2012). Interestingly, UV irradiation triggers another kinase, Atm- and Rad3-related protein (Atr), and induces a graded single pulse of p53 activation rather than pulsatile oscillation (Batchelor et al., 2011). The biological outcome of UV irradiation is apoptosis, which is different from the cases of γ-irradiation and synthetically controlled sustained p53. These results suggest that p53 dynamics (oscillatory, sustained and single graded pulse) can give rise to different cellular responses (cell cycle arrest/DNA repair, senescence and apoptosis, respectively).

### Pulsatile ERK: balancing proliferation and differentiation

ERK, one of the mitogen-activated protein kinases (MAPKs), also shows a variety of temporal patterns in response to growth factors. In PC-12 pheochromocytoma cells, epidermal growth factor (EGF) and nerve growth factor (NGF) activate ERK in a different temporal schedule. Upon EGF stimulation, MAPK is transiently activated in a pulsatile manner, which facilitates cell proliferation (Fig. 2D). By contrast, when cells are stimulated by NGF, MAPK activity shows sustained upregulation, which allows neuronal differentiation (Fig. 2D) (Marshall, 1995; Sasagawa et al., 2005; Santos et al., 2007). These observations indicate that the temporal patterns of ERK activation can control cell fate determination.

It also seems that the dynamic patterns of ERK activation depend on contexts and cell types. In cultured epithelial cell lines, treatment with EGF at a 1 ng/ml concentration triggers regulatory oscillations in ERK activity with a period of about 12 to 15 min (Shankaran et al., 2009). These dynamics can be monitored using a fusion protein, such as ERK-GFP, which allows visualization of the nuclear translocation of ERK in response to external stimulation. On the other hand, at physiological levels of EGF (in the order of pg/ml) or under normal culture conditions, temporal patterns of ERK activation show transient pulses in a more stochastic manner, with a firing rate ranging from 10 to 20 times per day (Albeck et al., 2013; Aoki et al., 2013). These stochastic pulses were visualized using a fluorescence resonance energy transfer (FRET) biosensor for ERK activity, EKAREV (Komatsu et al., 2011). The analysis of pulsatile ERK dynamics revealed that pulse frequency is modulated by culture conditions, such as EGF concentration and cell density, and highly correlates with proliferation rate. These data suggest that the mechanism of frequency modulation (Levine et al., 2013) in the ERK-MAPK signaling pathway plays an important role in controlling proliferation. The ERK-MAPK pathway includes a number of negative-feedback loops, but the network is too complicated to extract loops that might be essential for the emergence of ERK pulses. Furthermore, as the oscillation period is very short (about 15 min) compared with other slow processes, such as transcription and degradation, it is likely that faster enzymatic reactions, such as negative-feedback phosphorylation of the upstream activator Raf by activated ERK, might explain the pulsatile dynamics (Albeck et al., 2013; Aoki et al., 2013; Dougherty et al., 2005).

## Biological roles of ultradian oscillators: generating homogeneous and heterogeneous cell populations

The segmentation clock has the most intuitive benefit of oscillatory gene expression patterns. In the PSM, many genes are synchronously activated or repressed in a periodic manner, leading to the concordant triggering of differentiation cues in cell populations. As a consequence, cells enter the differentiation pathway with the same timing and generate tissue blocks (somites) of a certain size in a regulated and precise manner. If the synchronized state is disrupted by genetic manipulation, somite formation is severely disrupted, indicating an essential role for the synchronized oscillation in somite formation. Furthermore, when PSM cells are dissociated into single cells, oscillatory dynamics become unstable and out of sync, highlighting the importance of cell-cell communication for stable, synchronized oscillations (Maroto et al., 2005; Masamizu et al., 2006). In this context, Delta-Notch signaling seems to play a crucial role in synchronization, acting via cell-cell communication (Jiang et al., 2000; Riedel-Kruse et al., 2007; Niwa et al., 2011; Delaune et al., 2012).

Oscillations might also play a role in contributing to non-genetic heterogeneity, which results from a variety in gene expression patterns and is a consequence of genetic noise and/or gene regulatory networks rather than genomic mutations (Huang, 2009; Eldar and Elowitz, 2010). For instance, positive-feedback circuits, in concert with stochastic gene expression, can generate multiple states, such as the gene X-high and X-low populations shown in Fig. 1D, in a reversible manner. This might be advantageous for enabling a population of cells to make multiple cell fate decisions. In multipotent hematopoietic stem cells, two distinct populations with different expression levels of the stem cell marker Sca1 appear in a clonal population (Chang et al., 2008). Yet, both of these populations (Sca1-high and Sca1-low) can reconstitute self-renewing cultures with multipotency, suggesting a robust mechanism for the maintenance of stem cell pools, although transcriptome fluctuation is still under debate (Chang et al., 2008; Pina et al., 2012). This reconstitution takes more than one week and seems to be a slow relaxation process.

Hes1 oscillations also contribute to the heterogeneous differentiation responses of mouse ES cells. Due to oscillatory expression, Hes1 levels are variable among individual mouse ES cells, and Hes1-high cells tend to differentiate into mesodermal cells, whereas Hes1-low cells tend to differentiate into neural cells (Kobayashi et al., 2009). In the absence of Hes1, ES cells are prone to differentiate into neural cells more uniformly. Thus, the Hes1 ultradian oscillator contributes to heterogeneous properties of mouse ES cells (Kobayashi et al., 2009). When Hes1-high or Hes1-low mouse ES cells are sorted by FACS, the profile of Hes1 distribution is regenerated within 1 day. These fast kinetics are also seen in the case of Ascl1 and Hes1 in mouse neural progenitor cells (Imayoshi et al., 2013). Together, these examples demonstrate how ultradian oscillators contribute to the generation of non-genetic heterogeneity and give rise to multiple cell fates.

## Artificial control of oscillators: insights from optogenetic approaches

The accumulating evidence for ultradian oscillators in various biological contexts, as highlighted above, suggests a functional correlation between dynamic (oscillatory versus sustained) patterns of protein expression/activity and cellular responses. These observations have revealed sequential signaling cascades, from input signaling molecules to cell fate decisions, acting via oscillating molecules. However, these studies did not address how the pathway from oscillatory dynamics to cellular responses is regulated, and whether oscillatory dynamics are sufficient to initiate a particular cellular response.

One approach to resolve these questions involves generating artificial oscillations, without natural input signaling, and performing perturbation experiments in a genetically-targeted manner with spatiotemporal accuracy. Chemically inducible and tunable gene expression systems, such as Tet-On, are candidate methods, but the kinetics time scale of these methods is insufficient for the control of ultradian gene expression. Over the last decade, however, optogenetic systems in mammalian cells and vertebrate organisms have emerged as a successful approach for genetically targeting artificial gene expression in a precise spatiotemporal manner (Ausländer and Fussenegger, 2013; Lienert et al., 2014; Gautier et al., 2014). As light illumination can be applied with millisecond and sub-micron resolution, optogenetic approaches are more advantageous than classic pharmacological or genetic methods for fine-scale spatiotemporal resolutions. It is also worth noting that one advantage of the optogenetic approach, compared with pharmacological perturbation, is its modularity and specificity, because it is difficult to discover ideal pharmacological reagents that specifically target proteins of interest, whereas optogenetics works in a genetically more specific fashion.

The boom of optogenetics initially emerged in the field of neuroscience, in which genetically encoded opsins are used to control neuronal activities *in vivo* (Deisseroth, 2011), but optogenetic tools for the control of gene expression in other contexts are gradually developing. Currently available optogenetic modules for the control of gene expression are roughly categorized into two classes. One is the Phy-PIF system (see Box 1), which is responsive to red and infrared light (Shimizu-Sato et al., 2002; Levskaya et al., 2009; Müller et al., 2014). The other is a category of blue light (∼450-500 nm)-responsive systems, which are further divided into heterodimer (Yazawa et al., 2009; Kennedy et al., 2010; Polstein and Gersbach, 2012; Liu et al., 2012; Konermann et al., 2013) and homodimer (Wang et al., 2012; Motta-Mena et al., 2014) systems (see Boxes 2 and 3). One advantage of the blue light-responsive systems is that vertebrate cells endogenously synthesize chromophores of the photoreceptors, such as flavin adenine dinucleotide (FAD) and flavin mononucleotide (FMN). Indeed, these systems were successfully applied to zebrafish embryos and mice (Liu et al., 2012; Wang et al., 2012; Konermann et al., 2013; Motta-Mena et al., 2014). Such optogenetic systems (summarized in Table 1), which are further evolving, seem to be promising technologies for studying the significance of ultradian oscillators, as we discuss below.

**Table 1.**
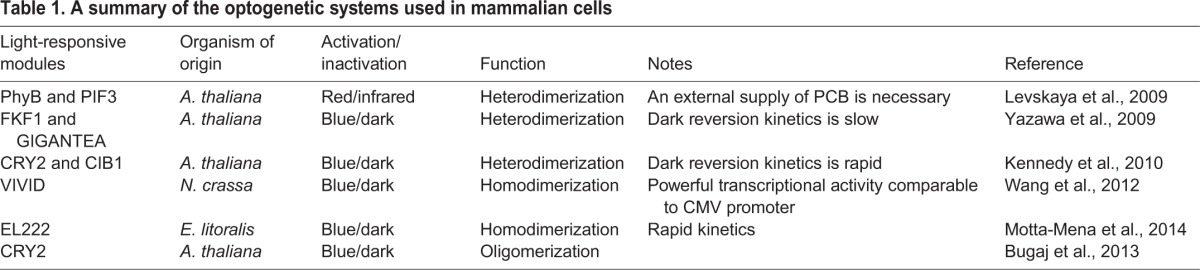
**A summary of the optogenetic systems used in mammalian cells**

Box 1. **The Phy-PIF system**

The Phy-PIF system, which is responsive to red and infrared light (Shimizu-Sato et al., 2002; Levskaya et al., 2009; Müller et al., 2014), consists of the *Arabidopsis thaliana* phytochrome B (PhyB) and the phytochrome interaction factor 3 (PIF3). In the presence of the chromophore phycocyanobilin (PCB), PhyB binds to phytochrome PIF3 and forms a heterodimer upon red light (∼650 nm) illumination. Infrared light (∼750 nm) illumination induces the dissociation of the PhyB-PIF3 protein complex and results in reversion within a time scale of the order of seconds (Levskaya et al., 2009). In mammalian cells, PCB is not synthesized and an external supply of this compound is therefore required, indicating a potential barrier for *in vivo* applications.

Box 2. **LOV domain-based systems**

LOV domain-based systems, which utilize the Light, Oxygen or Voltage (LOV) domain conserved in a wide variety of species, are divided into heterodimer (Yazawa et al., 2009; Polstein and Gersbach, 2012) and homodimer (Wang et al., 2012; Motta-Mena et al., 2014) systems. The first report of a LOV-based blue light-inducible gene expression system in mammalian cells was the FKF1-Gigantea system, which consists of a pair of proteins, FKF1 and Gigantea, which are derived from *Arabidopsis thaliana* (Yazawa et al., 2009). FKF1 contains a LOV domain that binds to blue light-sensitive flavin chromophores and changes conformation upon blue light stimulation, leading to the formation of FKF1-Gigantea heterodimers. One advantage of these systems is that vertebrate cells endogenously synthesize chromophores of the photoreceptors, such as flavin adenine dinucleotide (FAD) and flavin mononucleotide (FMN). Yazawa et al. (2009) fused a fragment of Gal4 protein to Gigantea and the VP16 transactivation domain to a fragment of FKF1 containing the LOV domain, resulting in a synthetic transactivator system. These factors are responsive to blue light, which induces the expression of genes of interest under the control of the UAS promoter. Moreover, replacing the Gal4 DNA-binding domain with different types of DNA-binding proteins, such as engineered zinc-finger proteins (ZFPs), enables activation of other promoter sequences, such as ZFP target sequences (Polstein and Gersbach, 2012). Blue light-induced dimers of LOV proteins dissociate in dark conditions due to the breakdown of the photo-adducts, and the kinetics of dark reversion varies across species and proteins. In the case of the FKF1-Gigantea pair, the decay proceeds very slowly, taking at least one day (Yazawa et al., 2009; Ito et al., 2012).

The photosensor Vivid (VVD), which is used by the fungus *Neurospora crassa* to control the circadian clock, is another LOV-domain protein that forms a homodimer upon blue light illumination. Wang et al. engineered a synthetic chimeric protein, termed GAVPO, which consists of a Gal4 DNA-binding domain, VVD with point mutations for reduced background activity in a dark state and a p65 transactivation domain (Wang et al., 2012). GAVPO can induce the transcription of genes fused downstream of UAS promoter sequences in mammalian cells, and its efficiency for transcriptional activation is comparable to the cytomegalovirus (CMV) promoter. As the dark reversion rate of VVD is about 5 h, this system (termed LightOn) can generate repeated pulses of gene expression patterns. Recently, EL222, another photosensor, which is derived from the Gram-negative bacterium *Erythrobacter litoralis*, was also reported to work as a synthetic blue light-sensitive transactivator in mammalian cells and in zebrafish embryos (Motta-Mena et al., 2014).

Box 3. **The CRY2-CIB1 system**

The CRY2-CIB1 system, which consists of *Arabidopsis* cryptochrome 2 (CRY2) and cryptochrome-interacting basic-helix-loop-helix (CIB1), shows rapid reversion kinetics in dark conditions (Kennedy et al., 2010). Using this approach, the light-induced translocation of proteins of interest can be stimulated in a repeated manner within a time scale of seconds. Liu et al. showed that this optogenetic module works not only in mammalian cells but also in zebrafish embryos (Liu et al., 2012), thus highlighting the possibility of *in vivo* applications. Furthermore, Konermann et al*.* combined this system with transcription activator-like effectors (TALEs) from *Xanthomonas sp.* and generated the light-inducible transcriptional effector (LITE) system, which consists of two fusion proteins, TALE-CRY2 and CIB1-VP64 (Konermann et al., 2013). Using the LITE system with a TALE that binds to the *Neurog2* promoter sequences, this system successfully activated endogenous *Neurog2* expression following blue light illumination. Interestingly, in the absence of CIB1, CRY2 protein can be induced to oligomerize upon blue light illumination (Bugaj et al., 2013). This phenomenon was applied to control the Wnt signaling pathway and activation of Raf (Bugaj et al., 2013; Wend et al., 2014).

**Fig. 3.**
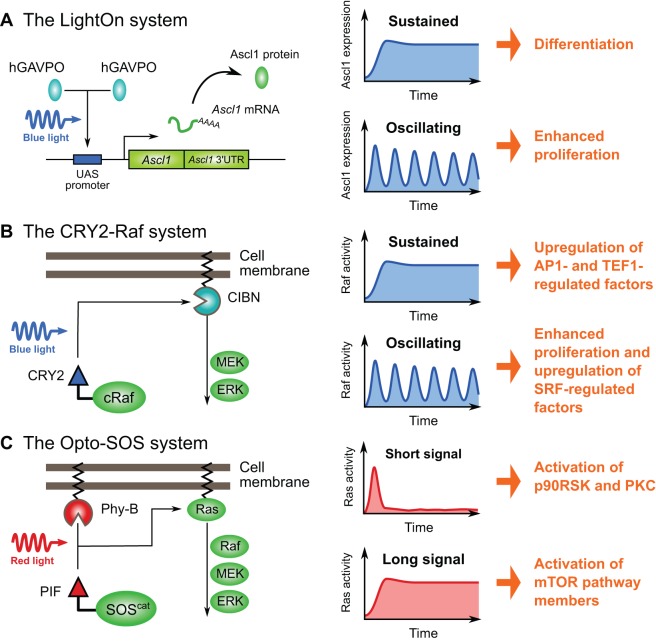
**Studying ultradian oscillations using optogenetic tools.** (A) The LightOn system has been used to study the functional roles of Ascl1 in neural progenitors (Imayoshi et al., 2013). In this system, GAVPO proteins form dimers upon blue light illumination, leading to the transcription of *Ascl1* fused downstream of the UAS promoter. Using this approach, it has been shown that sustained versus oscillating Ascl1 expression gives rise to different outcomes. (B) The CRY2-Raf system has been used to study the role of ERK pulses in proliferating cells (Aoki et al., 2013). Upon blue light stimulation, CRY2-cRaf rapidly associates with CIBN-KrasCT, which is anchored to the cell membrane. This association triggers activation of the MEK-ERK pathway. In the absence of light, CRY2-cRaf dissociates from CIBN-KrasCT, thereby inactivating the pathway. Using this method, it was shown that different schedules of ERK activation control the activation of different sets of genes. (C) The Opto-SOS system has also been used to study the MEK-ERK signaling cascade and confirmed that the dynamics of ERK activation indeed determine the downstream response (Toettcher et al., 2013). This system consists of the Phy-PIF system, which can be activated and inactivated by red and by near-infrared light illumination, respectively. When activated, PIF-SOS associates with the membrane-anchored Phy-B, resulting in activation of the Ras-MEK-ERK pathway. It is worth noting that the Opto-SOS system activates Ras, which is upstream of Raf, whereas the CRY2-Raf system directly controls Raf.

For example, Imayoshi et al. employed the codon-optimized GAVPO protein (Box 2) as a pulse generator of Ascl1 expression (Imayoshi et al., 2013) (Fig. 3A). Previous studies demonstrated that the proneural factor Ascl1 promotes cell cycle exit and subsequent neuronal differentiation (Bertrand et al., 2002; Wilkinson et al., 2013); however, it was also reported that Ascl1 directly activates the expression of genes involved in cell cycle progression in neural progenitor cells (Castro et al., 2011). Thus, how Ascl1 coordinates these contradictory functions remained unclear. Time-lapse imaging analyses showed that Ascl1 expression oscillates in neural progenitor cells but is sustained in differentiating neurons, suggesting that different expression dynamics could explain the contradictory functions of Ascl1 (Imayoshi et al., 2013). Indeed, analyses using the GAVPO system suggest that sustained expression of Ascl1 induces cell cycle exit and neuronal differentiation, whereas oscillatory expression of Ascl1 activates the proliferation of neural progenitor cells (Fig. 3A) (Imayoshi et al., 2013).

In the ERK signaling pathway, it has been suggested that the pulsatile frequency of ERK activity correlates with proliferation rate, depending on the culture conditions, such as EGF concentration and cell density (Albeck et al., 2013; Aoki et al., 2013). However, it was not clear whether the dynamic regulation of ERK pulses is sufficient to control cell proliferation. To address this issue, Aoki et al. generated a photo-inducible Raf system (CRY2-cRaf and CIBN-EGFP-KRasCT) based on the CRY2-CIB1 system (Box 3) (Kennedy et al., 2010), which could be used to assess the functional significance of ERK pulses during cell proliferation (Aoki et al., 2013). In this system, upon blue light stimulation, activated CRY2-cRaf associates with CIBN-EGFP-KRasCT, which is anchored to the cell membrane, and induces signaling from MEK to ERK. The reconstituted ERK pulses revealed that repeated activation of ERK promotes cell proliferation, whereas continuous activation does not (Fig. 3B). Furthermore, RNA-seq analysis following continuous or intermittent blue light illumination showed that different sets of genes are regulated in response to these different temporal schedules of ERK pulses. These direct approaches successfully demonstrated the functional significance of ERK pulses in cell proliferation.

The ERK signaling pathway can be also activated following recruitment of the guanine nucleotide exchange factor Son on sevenless (SOS) to the cell membrane. Taking this into consideration, Toettcher et al. combined a Phy-PIF module (Box 1) (Levskaya et al., 2009) with the catalytic segment of SOS and engineered a red light-tunable ERK activation system, termed Opto-SOS (Toettcher et al., 2013), which consists of a PIF-SOS fusion (tagged with YFP) and membrane-localized PhyB (PhyB-mCherry-CAAX) (Fig. 3C). Red light stimulation triggers the translocation of PIF-SOS to the cell membrane, thereby activating the Ras-MEK-ERK cascade. It was shown that platelet-derived growth factor (PDGF) activates both Ras-ERK and PI3K-Akt signaling, but the Opto-SOS system selectively activates the Ras/ERK pathway, enabling genetically targeted perturbation. Toettcher et al. applied the Opto-SOS system to analyze the downstream targets of Ras-ERK signaling and performed array-based proteomic screening upon PDGF stimulation or following pulsed (20 min) or sustained (120 min) red light illumination. The proteomic screening identified three types of downstream modules corresponding to the three distinct stimuli, suggesting that the dynamics of ERK activation indeed determine the downstream response.

In summary, it is clear that the dynamics of oscillatory proteins, such as Ascl1 in neural progenitor cells and ERK in proliferating cells, can be successfully re-constructed using optogenetic tools. As discussed above, different temporal schedules of light induction lead to different outcomes of cellular activities, such as proliferation and differentiation, supporting a functional role for ultradian oscillations in cellular decision making. Furthermore, the two complementary studies of the ERK pathway mentioned above that target different layers in the cascade support the usefulness of optogenetic technology for specific perturbation and demonstrate that optogenetic tools can shed light on the novel features of cellular oscillators as cell fate determinants.

## Perspectives

As we have highlighted above, molecular oscillators have been identified in various contexts, and recent studies, in particular those using optogenetic-based approaches, are beginning to elucidate the potential significance of ultradian oscillations. An important basic question is whether the signals observed in live imaging are really oscillations or stochastic pulses, in other words, whether they are derived from oscillatory systems or pulse generators. One possibility to distinguish oscillatory and pulsatile systems is to use response measurements of intracellular dynamics under the control of external temporal perturbation. Such experiments may be performed using optogenetic techniques.

Another unknown area is the mechanism by which the same factors regulate different gene sets depending on expression dynamics: oscillatory Ascl1 activates the proliferation of neural progenitors, whereas sustained Ascl1 induces cell cycle exit and neuronal differentiation (Imayoshi et al., 2013); continuous or intermittent ERK activation regulates different gene sets (Aoki et al., 2013). To date, many studies of ultradian oscillators have used chemicals, growth factors or radiation in order to temporally perturb the oscillating systems. As these perturbations disrupt not only the dynamics of ultradian oscillators but also the topology of the network of interest, it has been difficult to conclude whether ultradian oscillations or sustained dynamics of a single key regulator are sufficient to elicit a certain biological event. However, newly evolving techniques described here, such as novel biosensors and optogenetic tools, are providing deeper insights into how cells interpret different dynamics of gene expression.

Lastly, it is important to examine the functional role of ultradian dynamics *in vivo*. In the case of p53, Hamstra et al. observed oscillatory dynamics at the tissue level by *in vivo* bioluminescence imaging (Hamstra et al., 2006). Another example is the observation of single-cell dynamics of ERK pulses in epithelial cells of the mammary gland (Aoki et al., 2013). Hes1 and Ascl1 oscillatory expression has also been observed in slice cultures (Imayoshi et al., 2013), and oscillation dynamics of the segmentation clock have been examined in PSM explant cultures (Masamizu et al., 2006; Aulehla et al., 2008). However, with the exception of the segmentation clock, oscillatory expression is not synchronized at the population level, and its phase control between neighboring cells in tissues also remains to be analyzed. Live imaging at single cell resolution in *in vivo* tissues is required to address this issue. Moreover, the optogenetic applications shown above were carried out in *in vitro* experiments. To bring optogenetic approaches to embryos *in vivo*, many problems still need to be overcome, including the delivery of light into deep tissues, illumination with precise spatial resolution and the generation of transgenic animals carrying the optogenetic systems in a tissue-specific manner. Thus, *in vivo* application is still highly challenging, but resolving these issues by *in vivo* imaging together with optogenetic manipulation may enhance our understanding of biological significance of ultradian dynamics and offer novel approaches for therapeutic applications.
